# 

**Published:** 1897-05

**Authors:** 


### THE
### AMERICAN JOURNAL
OF
### DENTAL SCIENCE,

EDITED BY

F. J. S. GORGAS, M. D., D. D. S.

RICHARD GRADY, M. D., D. D. S.

VOLUME XXXI—THIRD SERIES.

LIBRARY
SURGEON GENERAL'S OFFICE
FEB. 11.-1901
168864.

BALTIMORE:
SNOWDEN & COWMAN MFG. CO.

LONDON:
TRUBNER & CO., PATERNOSTER ROW.
1898.

### Index to Vol. XXXI---Third Series.

A Practical Treatise on Artificial Crown and Bridge-work... 46
An Empty Pulp Canal........ ....... ... ....... ...... 46
A Professional Warning ........................................ 77
American Dental Association.................................. 85
A Light Artificial Denture............ ..... .............. 88
Absorbed Ridge.................................................. 89
An Impression and Its Tests.............. ... ............ 90
Ambidexterity.................................................... 10[illegible]
An Apparatus for Drainage............. ................ 115
A Litter of Supernumary Teeth............................ 139
A Useful Flux.. ..... ......:................................ 144
Anti-Extraction .. ........................... .............. 187
An Interesting Case of Membranous Stomatitis............. 190
Adhæsol .......................................................... 191
A Cure for Hiccough ........... ... ..... ............. 240
Acetanilide as a Dressing.............. ............... 284
A Simple Way of Putting on Metal Backings for Vulcanite Work Without Soldering.............................. 285
A Good Law............ .. ..................... 285
A New Hemostatic................................. 287
A Hint on Extracting....................... ......... 288
Ammonol......... ............... ................ 288
A Manual of the Injuries and Surgical Diseases of the Mouth, Face and Jaws............. ....... ............... 331
A New Wart Cure.............................................. 335
A Colored Lady Doctor........... ..... ............... 379
A Good Cement............................ .. ........... 379
A Lake of Petroleum........................................ 380
A Useful Disinfectant................................. ..... 420
Appendicitis.. ........ ......................... .... 524
Antiseptics and Germicides in Dentistry .. ............... 529
American Medical Association ............................ 571
Blood-poisoning after Tooth Extraction ..... .............. 240
Blood Stains........ ................ ............... 477
Be Cleanly................................................... 528
Competive Examinations for Scholarships................... 1
Compend of Dental Pathology and Therapeutics........... 45
Cocaine......................................................... 57
Cleaning the Teeth ..... ..... ............... 59
Compound Cement and Amalgam Filling.................. 60
Carbolate of Iodin.......................................... 92
Care of the Mouth in Relation to Prophylaxis of the Digestive Tract.......................... ........... 97

Cataphoresis in the Removal of Vital Pulps followed by Immediate Root Filling ........ 135
Catching Cold ........ 142
Cocain Solution ........ 239
Cataphoresis ........ 275
Children's Teeth as Indicators of Disturbed Nutrition ........ 363
Character in our Features ........ 398
Drawing Room ........ 48
Dental Chemistry and Metallurgy ........ 86
Dental Operations During Pregnancy ........ 87
Difficult Partial Impressions ........ 93
Dental Hygiene ........ 109
Dental Laws and Their Enforcement ........ 121
Dr. Samuel J. Hayes ........ 185
Discussion on Soldering ........ 227
Difficult Partial Impressions ........ 238
Different Method of Heating Guttapercha ........ 240
Discussion on Local Anesthesia ........ 286
Don't Use Plain Teeth ........ 333
Diamond Drills, How to Use ........ 336
Dr. DeWilton Snowden Dead ........ 374
Dr. Evans ........ 376
Dental Examiners of Maryland. Validity of Their Appointment and Their Actions Questioned in a Suit Instituted ........ 422
Dr. William S. Mcdowell ........ 423
Dr. S. C. Wilkerson ........ 426
Deafness ........ 432
Drainage and Irrigation of the Antrum ........ 452
Discolored Teeth ........ 574
Eucaine ........ 91
Economy in Dental Practice ........ 149
Electricity and Dentistry ........ 325
English People's Teeth ........ 381
Evil Effects of Mercury ........ 393
Economics of Dentition ........ 441
Extracting Teeth ........ 446
Experience ........ 479
Fifth Annual Commencement of the University of Buffalo, Dental Department ........ 40
Function of the Upper Third Molar ........ 94
Filling Roots ........ 119
Facts About Steel ........ 220
Filling Frail Teeth ........ 236
Faulty Nutrition ........ 378
For Inflamed Eyes ........ 379
Formagen ........ 450

For Burns........ 527
Four Classes of Dentists ........ 534
Fastidious Operating........ 576
Galvanic Action in the Mouth........ 92
Gold Fillings Inserted while Cavity Filled with Saliva........ 474
How Can We Educate the Public........ 108
How and Why we Treat and Fill Root-Canals—Our Way........ 175
Hearing Restored after Twenty-five Years........ 192
How to Establish a Dental Practice........ 385
Hints ........ 477
Habits and Products of Germs........ 544
Hints.—By a Lazy Man........ 572
Influence of Climate and Diet on Teeth........ 25
Incidents in Office Practice........ 76
Impressions of Modeling Compound........ 85
Improved Porcelain Bridge........ 189
Incisor or Cuspid Crown ........ 192
Illinois State Dental Society, 1897........ 193
Insensibility to Pain........ 337
In Memorium........ 424
Ingratitude........ 480
Is the Dental Profession Crowded ........ 503
Kaiserling's Method for the Preservation of Specimens with their Natural Colors........ 287
Light in Vacuum Tubes........ 112
Listerin........ 118
Life and Death in Emotions........ 188
Lactic Acid in Pyorrhœa........ 334
Medical Dictionaries........ 44
Metal Pins........ 140
Medical Superstitions........ 287
Massage of the Gums ........ 384
Making Partial Dentures........ 526
New Method of Administering Chloroform........ 138
National Association of Dental Faculties........ 177
National Association of Dental Examiners ........ 185
No Sympathy for Non contributors........ 191
Nevermind the Other Fellow........ 240
Northern Illinois Dental Society........ 283
Necrosis of Hard Palate ........ 481
New Operative Method for the Radical and Rapid Cure of Chronic Empyemia of Maxillary Sinus........ 488
Oral Surgery: Theory and Results........ 63
Oxychloride of Zinc ........ 308
Office Drowsiness ........ 431
On Certain Difficulties of Diagnogis........ 505

Oral Hygiene .... 551
Pyorrhea Alveolaris—What do We Really Know of it .... 16
Porcelain Fillings .... 19
Potter's Materia Medica, Pharmacy and Therapeutics .... 45
Pulpless Teeth Again—Repeated Dressings .... 54
Pearson's Local Anæsthetic .... 138
Pulp Devitalization in the Teeth of Children .... 139
Plantation of Teeth .... 155
Pyrozone .... 225
Pitiable Condition of a Girl from Use of X-Rays .... 236
Point of Contact .... 272
Practical Things with Illustrations .... 289
Pecularities of Woman .... 332
Practical Things in Dental Practice .... 370
Prevention of Tartar .... 380
Popular Errors Concerning Snakes .... 383
Preserving Gutta-Percha .... 384
Prominent Causes of Failure in the Use of Electricity in Dentistry .... 468
Proposed Dental Law .... 562
Prescription Writing .... 573
Queer Things about Mankind .... 464
Questions Given to Applicants for Certificates to Practice Dentistry in Maryland .... 568
Richardson's Mechanical Dentistry .... 43
Resolutions in Memory of Dr. Frank Abbott .... 137
Root Peforation—A New Method of Treatment .... 237
Rules for the Care of the Eyes .... 238
Resection and Reproduction of the Maxillæ .... 263
Report of the Committee on Necrology of the National Association of Dental Faculties, at Old Point Comfort .... 329
Removal of Blood Stains .... 383
Report of a Case of Carcinoma of the Buccal Mucous Membrane with Microscopic Exhibit .... 399
Rontgen Rays .... 475
Some Established Principles in Prosthetic Dentistry .... 31
Sensitive Dentine .... 35
Some Points About Artificial Palates .... 37
Swaging Plates .... 87
Space in Filling .... 89
Soldering .... 130
Separating Teeth .... 163
Some Points in the Ether-Chloroform Controversy .... 209
Suicide by Taking Cocain .... 239
Singular Death from Chloroform at Yardley .... 310
Sins .... 333

Successful Method of Filling Fast Decaying Teeth of the Young and Anemic........ 414
Shall the Dentist Advertise........ 490
Stimulants and Sedatives........ 518
Saving an Old Tooth ........ 520
Setting Bridges ........ 528
Shall the Dentist Advertise? ........ 575
Students Practising Dentistry........ 576
The Enamel Organ........ 9
The National Association of Dental Examiners........ 39
The Chicago Dental Society........ 40
The Empire Dental Sterilizer ........ 41
The American Text Book of Prosthetic Dentistry........ 42
The destiny of the Third Molar ........ 48
The Reason of the Faith that is in Me ........ 49
Two Interesting Cases of Capped Pulps........ 52
The Care of the Teeth........ 83
The National Association of Dental Examiners........ 84
Transactions of the New York Odontological Society for 1895. 86
To Prevent Rust ........ 93
To Diagnose Pulpitis........ 95
To Relieve Sensitiveness After Grinding a Tooth........ 96
Three Pathological Cases........ 126
Treating Pulpless Teeth........ 141
The Sense of Touch ........ 143
The Antiseptic Properties of Saliva ........ 143
The Patient ........ 145
The Study of Anatomy........ 168
Tuberculosis of the Alveolar Process........ 190
Teeth in Relation to the Ear, Nose and Throat ........ 192
Trichloracetic Acid as a Solvent of Necrosis........ 222
The Bacteriology of Baldness ........ 236
The Clinic ........ 241
To What Extent and When are We Justified in Using Cataphoresis? Is There Danger of Injuring the Dental Pulp or Other Tissues by its Use? ........ 243
Treatment of Pulpless Teeth........ 260
To Open Pulp Chambers of Teeth Affected with Pericementitis........ 288
The Preparation of Dental Alloys and Cements........ 299
The Physiology of Cataphoresis........ 303
The National Association of Dental Examiners........ 312
The National Association of Dental Faculties ........ 317
The American Text Book of Prosthetic Dentistry ........ 326
The Arrest of Hemorrhage........ 332
The New Dental Law in Pennsplvania........ 334

The Dental Student ... 334
To Keep Spittoon Clean ... 336
The Entrance Examinations in Dental Colleges ... 339
The Possibilities of Prosthetic Dentistry ... 343
Treatment of Abscess ... 349
The Century's Progress in Chemistry ... 354
Trichloracetic Acid as a Solvent of Necrosis ... 362
The Dentist ... 368
The Micromotoscope ... 382
To Cement Metal to Glass ... 384
The Etiology of Dental Caries ... 402
The Bleaching of Discolored Teeth ... 406
The Late Drs. Frank Abbott and L. G. Ingersoll ... 428
The American Text Book of Operative Dentistry ... 429
The Standard of the Natural Board of Dental Examiners ... 433
Tumors Resulting from Septic Pulp of Teeth ... 454
Treatment and Filling of Root Canals ... 458
The Care of Vulcanizers ... 475
The Treatment of Teeth Having Putresent Pulps ... 483
The Adaptation and Retention of Artificial Dentures ... 494
The Fracture of Platinum Pins in Teeth and the Cracking of Teeth in Soldering ... 497
Treatment of Protrusion of the Lower Teeth ... 501
The Care of Vulcanizers ... 525
The Natural Law of Cure ... 539
To Remove Enamel Previous to Crowning ... 547
The Educational Features of the Dental Association ... 549
The Use of Soft Foil ... 557
Unnecessary Sacrifice of Dental Pulps ... 4
Union of the American and the Southern Dental Associations ... 253
Use Your Judgment ... 392
Urotropin ... 472
Varnishing Cavities ... 114
Value of Meat Extracts ... 477
Value of Examination of the Mouth in the Choice of a Nurse. 487
What to Try ... 381

### Communicated.

### THE NATIONAL ASSOCIATION OF DENTAL EXAMINERS.

The fourteenth annual session will be held at Old Point Comfort, Va., commencing Friday morning, July 30th, at 10 a. m., and continuing in session Saturday, July 31st, and Monday, Aug. 2nd. The sessions will be held in the Hotel Chamberlain.

The hotels "Hyegia" and "Chamberlain" will give rates of $2.50 per day two in a room, $3.50 per day one in a room.

Fares on all the Trunk lines one full fare and one third, good for the sessions of the American and Southern Dental Associations.

The Old Dominion S. S. Co., Pier 26 North River will sell excursion tickets including meals and berths, $11.20, sailing every day 3 p. m., from Thursday, July 29, using the name of the Secretary.

Let every State send its delegates.

CHARLES A. MEEKER, D. D. S., Sec'y,
29 Fulton Street, Newark, N. J.

### THE CHICAGO DENTAL SOCIETY.

CHICAGO, April 13th, 1897.

*American Journal Dental Science:*

The following is the list of officers of the Chicago Dental Society for 1897-98 elected at the annual meeting held in Columbus Memorial Building, Tuesday evening, April 6th, 1897: President, A. H. Peck; 1st vice-president, D. M Gallie; 2nd vice-president, G. T. Carpenter; recording secretary, Geo. B. Perry; treasurer, E. D. Swain; member Board of Directors, J. N. Crouse; Board of Censors, D. M. Cattell, A. W. McCandless, J. J. Whaley.

Yours truly,

GEO. B. PERRY,

Corresponding Sec'y.

### FIFTH ANNUAL COMMENCEMENT OF THE UNIVERSITY OF BUFFALO, DENTAL DEPARTMENT.

BUFFALO, N. Y., May 1, 1897.

*Editor The American Journal Dental Science:*

The Dental Department of the University of Buffalo held its fifth annual commencement exercises, in connection with the Departments of Medicine and Pharmacy, in Music Hall, Buffalo, on the evening of April 27th, when the Chancellor conferred upon the following named students the Degree of Doctor of Dental Surgery.

The whole number of students matriculated for the term was 222. The number of seniors was 70. The junior class numbered 70. The freshmen numbered 82.

CLASS IN DENTISTRY.

Bertram John Baker, Jarvis Barraclough, Alfred Milton Borton, William Penn Brinton, Colin Cameron, Alfred Rayson Cook, Jay Herbert Dower, John Vosburgh Flaherty, Frank Arthur Gough, Frederick Dodge Greene, Leverett Lorenzo

Hamilton, Perry Frederick Hepp, Abram Lauder Hipwell, Henry Hamilton Ketcham, William Charles Luetti, Harry Benjamin Lyon, William Hyde Marks, William Spence McFarlane, Frank Chance McPhcrson, William Drysdale Moss, Robert Muir, Leonard Earl Noble, Seth Theodore Paine, Charles Henry Plumstead, Harry Pond Rawson, Wilbur Salathiel Rose, Clarence Levi Roys, Ph. B., Daniel George Simmonds, Howard Eugene Stone, Harry Nedder Tefft, Frank Hicks Underwood, Charles Henry Whitlock, Melrose Williams Wright, George Daw Wood, Harry Eddy Barnhart, Frederick Sherwood Belding, Warren Joseph Boyd, Alfred Byron Cairnes, Harry More Clark, Charles Stephen Denel, James Vernon Faulkner, Ferdinand John Freitag, Francis Leonard Greene, Ph. B., Beverly Chew Guile, George Tyler Head, M. D., George Taylor Hickelton, Carey Elijah Janes, Van Albert Lucy, George Thomas Lord, Arthur Bastedo Magee, Charles Jason McClure, John Frederick McPhee, Henry Stuart Merkley, Theodore Perkins Moyer, Frederick Bryant Niles, Henry Richard O'Brien, Charles Bicknell Payne, William Thomas Potter, Louis St. George Rickards, Samuel Rowan, Rolla Sabine, Frank Walton Smith, John Marcus Tanzer, Anna Mathilde Thrane, Clarence Renwood Waldron, Harry Lawson Whipple, Edward Arthur Wood.

### Editorial, Etc.

### To Readers and Correspondents.

Communications intended for publication, and exchange journals and papers, should be addressed to the Editor of The American Journal of Dental Science, No. 845 N. Eutaw St. Baltimore, Md. All letters relating to the business of the Journal, or containing cash remittances, to be directed to Snowden & Cowman Mfg. Co., 9 W. Fayette Street, Baltimore, Md. Articles for publication should be received by the Editor at least two weeks previous to the first of the month on which the number in which it is designed for them to appear is issued.

### COMMUNICATED.

The National Association of Dental Examiners .... 39
The Chicago Dental Society .... 40
Fifth Annual Commencement of the University of Buffalo, Dental Department .... 40

### EDITORIAL, ETC.

The Empire Dental Sterilizer .... 41

### BIBLIOGRAPHICAL.

The American Text Book of Prosthetic Dentistry .... 42
Richardson's Mechanical Dentistry .... 43
Medical Dictionaries .... 44
Potter's Materia Medica, Pharmacy and Therapeutics .... 45
Compend of Dental Pathology and Therapeutics .... 45
A Practical Treatise on Artificial Crown and Bridge-work .... 46

### MONTHLY SUMMARY.

An Empty Pulp Canal .... 46

Unnecessary Sacrifice of Dental Pulps .... 47

The destiny of the Third Molar .... 48

Drawing Room .... 48

☞ The American Journal of Dental Science will contain fifty pages of reading matter in each number, making a volume of about Six Hundred pages,

EXCLUSIVE OF ADVERTISEMENTS.

